# Essentials of Insulinoma Localization with Selective Arterial Calcium Stimulation and Hepatic Venous Sampling

**DOI:** 10.3390/jcm9103091

**Published:** 2020-09-25

**Authors:** Ken Zhao, Nihal Patel, Kopal Kulkarni, Jonathan S. Gross, Bedros Taslakian

**Affiliations:** Division of Vascular and Interventional Radiology, 660 1st Avenue, Room 318, New York University School of Medicine, New York, NY 10023, USA; Ken.Zhao@nyulangone.org (K.Z.); Nihal.Patel@nyumc.org (N.P.); kopal.kulkarni@nyumc.org (K.K.); Jonathan.Gross@nyumc.org (J.S.G.)

**Keywords:** insulinoma, localization, selective arterial calcium stimulation, interventional radiology

## Abstract

Insulinomas are the most common functional pancreatic neuroendocrine tumor. Most insulinomas can be localized non-invasively with cross-sectional and nuclear imaging. Selective arterial calcium stimulation and hepatic venous sampling is an effective and safe minimally-invasive procedure for insulinoma localization that may be utilized when non-invasive techniques are inconclusive. The procedure’s technical success and proper interpretation of its results is dependent on the interventional radiologist’s knowledge of normal and variant pancreatic arterial perfusion. Accurate pre-operative localization aids in successful surgical resection. Technical and anatomic considerations of insulinoma localization with selective arterial calcium stimulation and hepatic venous sampling are reviewed.

## 1. Introduction

Insulinomas are the most common functional pancreatic neuroendocrine tumor, with an incidence of 4 cases per million person-years [1]. They are most common in middle-aged patients and are often sporadic, but can be associated with Multiple Endocrine Neoplasm (MEN-1), Von Hippel-Lindau (VHL), Neurofibromatosis type 1 (NF1), and tuberous sclerosis. Approximately 10% of cases consist of multiple tumors, less than 10% of tumors are malignant, and 5–10% of all insulinomas are associated with MEN-1 syndrome [1]. These latter MEN-1 tumors are usually multiple and can be malignant in up to 25% of cases [2].

Hyperinsulinemia secondary to insulin secretion by insulinomas results in Whipple’s triad of (1) symptomatic hypoglycemia, especially after fasting/exercise, (2) serum glucose <40 mg/dL, and (3) symptomatic relief after glucose administration. Hypoglycemic symptoms include neuroglycopenic symptoms (confusion, visual changes, amnesia, altered consciousness, headache, and seizures) and autonomic symptoms (sweating, weakness, palpitations, tremor, paresthesias, and hunger). In healthy people, post-absorptive levels of plasma glucose stay within a narrow range (about 60 to 100 mg per deciliter) despite the intermittent ingestion of food [3]. However, insulinomas can result in hypoglycemia during both fasting and, less commonly, postprandial states [3,4,5,6].

Accurate biochemical diagnosis of endogenous hyperinsulinemic hypoglycemia must first be obtained. An endocrinology consultation is required, given the diagnostic nuances and implications of potentially invasive insulinoma localization and major surgery. Diagnosis is typically established by supervised fasting up to 72 h with concurrent measurements of beta-cell polypeptides (insulin, C-peptide, and proinsulin), detecting up to 99% of insulinomas [7,8]. This allows documentation of clinical symptoms in relation to hypoglycemia and inappropriate hyperinsulinemia in the setting of hypoglycemia [9]. Additionally, elevated C-Peptide and pro-insulin levels have been shown to have greater diagnostic accuracy for the diagnosis of endogenous hyperinsulinism than insulin levels [10]. Other etiologies of adult-onset endogenous hyperinsulinemic hypoglycemia, such as noninsulinoma pancreatogenous hypoglycemia syndrome, should be considered. Inappropriate administration of insulin secretagogues or exogenous insulin must be excluded by careful history taking, medication review, and possible plasma secretagogue assay.

Surgical management is first-line for treatment of symptomatic insulinoma and is highly effective, with a reported 5-year disease-free rate of 100% [11]. Non-surgical techniques, such as transcatheter embolization, ethanol ablation, and radiofrequency ablation, are not yet well-established. Of these, ethanol ablation by endoscopic ultrasound (EUS) guidance is the most reported and achieves symptomatic relief in 85% of cases [12]. Medical options for symptomatic management in patients who are not surgical candidates include diazoxide and octreotide, both of which decrease insulin secretion.

In order to successfully resect the tumor and minimize loss of normal pancreatic tissue, the insulinoma must be identified and accurately localized. However, insulinomas can be difficult to detect with non-invasive imaging. They are often small at the time of presentation and may occur in any part of the pancreas. When non-invasive imaging fails to identify a suspected insulinoma, selective arterial calcium stimulation with hepatic venous sampling should be considered [13,14,15].

The goals of this article are to review relevant pancreatic vascular anatomy, including anatomic variants, to describe the calcium stimulation test and to offer tips for procedural success, and to discuss the interpretation of the test’s results.

## 2. Non-Invasive Pre-Operative Localization

Insulinomas may be difficult to localize by intraoperative inspection, as 9–23% of tumors cannot be found on palpation during laparotomy [16,17]. Pre-operative localization can maximize the likelihood of finding the tumor intraoperatively and successfully excising it (Table 1) [16,18].

Transabdominal ultrasound (US) is occasionally the first non-invasive imaging modality used for insulinoma localization since it is widely available and relatively inexpensive. However, because pancreatic insulinomas are typically <2 cm at time of presentation and bowel gas or abundant adipose tissue can limit visualization of the pancreas, ultrasound is limited in this setting and localizes the tumor in 9–63% of cases [19,20].

**Figure 1:**
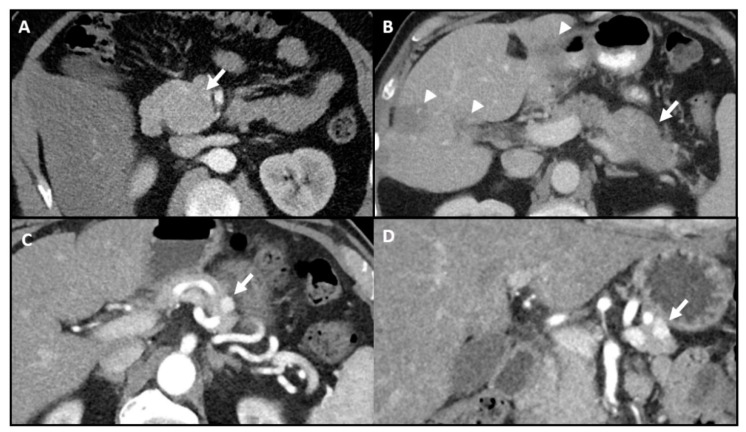
Variable appearance of pancreatic insulinomas on contrast-enhanced computed tomography (CT). (**A**) Axial image shows a 5.5 cm isodense insulinoma (arrow) in the uncinate process. (**B**) Axial image shows hypodense, malignant insulinoma within the pancreatic tail and liver metastases (arrowheads). (**C**,**D**) Axial and coronal images show an avidly enhancing 1.2 cm insulinoma in the pancreatic tail. All were proven to be pancreatic insulinomas by surgery or biopsy.

Cross-sectional imaging with contrast-enhanced computed tomography (CT) is often first line for localization of pancreatic insulinomas. Pancreatic insulinomas are typically well-circumscribed and hyper-vascular with greatest enhancement during the arterial phase [34]. The localization rate with CT ranges from 63–94%, as the imaging appearance is variable and lesions are predominantly small at presentation (Figure 1) [22,23,24]. The aforementioned upper localization rate of 94% can be attributed to the improved spatial resolution and decreased slice thickness of modern multidetector CT [24].

Contrast-enhanced magnetic resonance imaging (MRI) has a reported insulinoma detection rate of 80–90%, which is similar to modern thin-slice multidetector CT [25,26]. Unlike CT, MRI does not utilize ionizing radiation nor iodinated contrast, and is thus appropriate for young patients or those with renal impairment. MRI’s advantages are tempered by its high cost and long image acquisition times. On MRI, insulinomas are typically hypointense relative to normal pancreatic parenchyma on pre-contrast T1-weighted imaging and demonstrate hyper-enhancement on the arterial phase [25,34]. The addition of diffusion-weighted MRI may aid the detection of small insulinomas, especially those without hypervascular enhancement [26,35].

**Figure 2:**
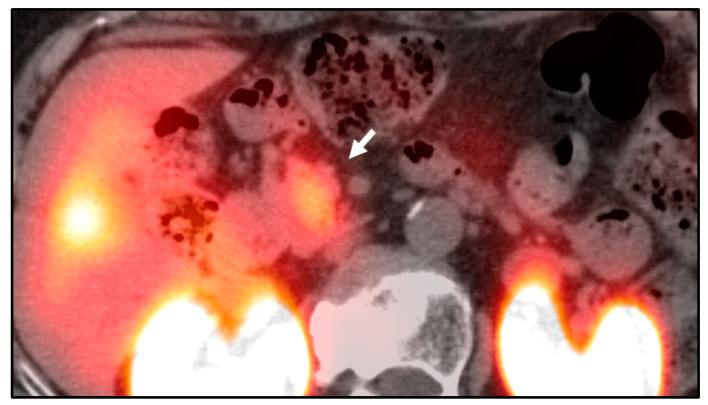
Occult insulinoma localized by ^111^In-Octreotide. Axial SPECT/CT fusion demonstrates focal intense uptake within the uncinate process (arrow). A corresponding insulinoma was confirmed by biopsy. This insulinoma was occult on prior contrast enhanced magnetic resonance imaging (MRI).

Nuclear imaging modalities are becoming increasingly prevalent for detecting pancreatic neuroendocrine tumors, including insulinomas. The somatostatin type-2 receptors expressed by neoplastic insulinoma cells can be targeted by radiotracers such as ^111^In-Octreotide and ^111^In-Pentreotide (Figure 2). Somatostatin receptor imaging has demonstrated a sensitivity of 62–85% for detection of pancreatic neuroendocrine tumors [27,28]. However, somatostatin receptor imaging has a lower sensitivity of 47–60% for the detection of pancreatic insulinomas, possibly due to decreased expression of somatostatin type-2 receptors [18,36,37]. A newer somatostatin analog radiotracer, ^68^Ga-DOTATATE, is hypothesized to have greater affinity for somatostatin type-2 receptors. However, reports have been conflicting, with insulinoma localization rates ranging from 32 to 90% [29,30].

Novel radiotracers targeting glucagon-like peptide 1 receptors (GLP-1 R), which are expressed by benign insulinomas at a high density, are currently under investigation and show promising results [38]. For instance, ^68^Ga-DOTA-exendin-4 PET/CT recently demonstrated a sensitivity of 93.9% for detection of benign insulinoma [39].

**Figure 3:**
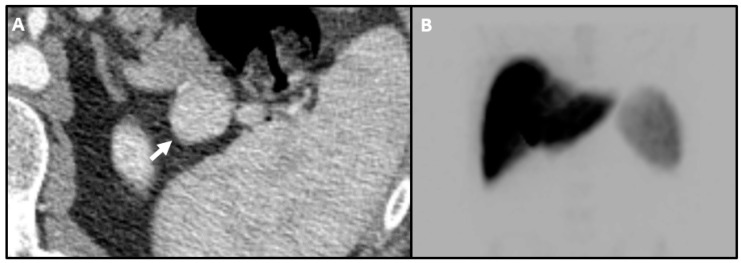
Mass adjacent to pancreatic tail. (**A**) Axial CT image shows an ambiguous enhancing mass (arrow). It is unclear whether this mass is intra- or extra-pancreatic and splenule was raised as a possibility. (**B**) Frontal planar ^99m^Tc-Sulfur Colloid image without focal uptake in the region of the pancreatic tail, making splenule unlikely. This mass was surgically excised and confirmed to be an insulinoma.

Nuclear imaging can also be used for troubleshooting. Splenules, accessory foci of normal splenic tissue, are known mimickers of pancreatic tail lesions and can make the imaging diagnosis and intraoperative search for pancreatic lesions more difficult [40,41]. On MRI, splenules should exhibit the same imaging features on every sequence as the spleen, but MRI is less helpful when a spleen is not identified [42,43]. ^99m^Tc-Sulfur Colloid can differentiate splenules from insulinomas based on uptake pattern, which should be high in splenic tissue and low in pancreatic neoplasms (Figure 3) [44].

Cross sectional imaging may be obtained for pre-procedural evaluation of disease extension or metastatic spread. The anatomic characterization provided also aids planning of potential surgery or invasive localization procedures. Nuclear imaging can also be used to assess extent of disease.

## 3. Invasive Pre-Operative Localization

If non-invasive imaging for localization of insulinoma is negative or inconclusive, invasive pre-operative localization with EUS and/or the selective arterial calcium stimulation test (SACST) should be considered. EUS is very effective for detection of insulinomas in the pancreatic head with a reported localization rate of up to 92.6% [31]. However, if the insulinoma lies in the pancreatic body or tail, the sensitivity drops to 78.9% and 40%, respectively [31]. Additionally, some insulinomas are completely isoechoic and cannot be visualized on EUS regardless of their location in the pancreas [45]. Reported risk factors for falsely negative EUS include low body mass index, female sex, and young age [46]. If an accessible lesion is visualized, biopsy for tissue sampling may be obtained during EUS.

Arteriography alone without calcium stimulation or venous sampling used to be considered the “gold standard” for insulinoma localization [47]. However, it is highly dependent on operator skill and expertise, with reported sensitivities ranging from 29–64% [21,48,49]. Due to its unreliability and improvements in non-invasive imaging, arteriography alone is now seldom used for insulinoma localization.

The selective arterial calcium stimulation test is a minimally invasive preoperative localization method and will be discussed in detail in later sections [50]. SACST is typically indicated in the setting of biochemically confirmed hyperinsulinemic hypoglycemia when (1) there is no identifiable focal lesion on non-invasive imaging, (2) there are multiple possible functional lesions, or (3) there is a focal lesion but additional functional confirmation is necessary. The second scenario is especially applicable to patients with MEN-1, as the functional insulinoma may need to be identified amongst additional synchronous nonfunctional pancreatic neuroendocrine tumors [51].

In cases where the pancreatic insulinoma can be confidently identified on non-invasive imaging, the SACST need not be performed. For example, a well-delineated lesion with typical findings on contrast-enhanced cross-sectional imaging and concordant nuclear imaging findings can be confidently diagnosed as insulinoma. Concordant findings on cross-sectional imaging and EUS should also be considered confidently diagnostic, as the diagnostic sensitivity for combined thin-section CT and EUS for insulinoma was found to be 100% [24].

## 4. Intraoperative Localization

The surgeon may localize the tumor intra-operatively. Intraoperative inspection and palpation of the pancreas has a sensitivity of ~77–91% for insulinomas, though some of these tumors had already been detected on pre-operative testing [16,17]. The surgeon may utilize intraoperative ultrasound, which is particularly useful for lesions that are proximal to the pancreatic or biliary duct and would otherwise be more difficult to find by inspection/palpation alone [52]. Intraoperative ultrasound is more sensitive than palpation alone and successfully localizes insulinomas in ~91–93% of cases [17,52,53,54,55]. In cases of unsuccessful pre-operative localization, an experienced surgeon may successfully localize the tumor intraoperatively. The surgeons in one study successfully detected the tumor in all 6 patients without pre-operative localization, and, in another study, successfully detected the tumor in all 5 patients without pre-operative localization [11,17].

Intraoperative blood glucose or insulin monitoring can help confirm successful insulinoma resection. This confirmation is particularly relevant in the setting of multiple functional tumors or when resection margins are unclear. Venous blood is drawn before and after tumor removal. Once all tumor tissue is successfully removed, blood glucose should increase by approximately 30 mg/dL within 30 min and plateau after about 60 min [56]. Correspondingly, the blood insulin level should sharply fall within 30 min after tumor resection [57].

## 5. Selective Arterial Calcium Stimulation and Hepatic Venous Sampling

**Figure 4:**
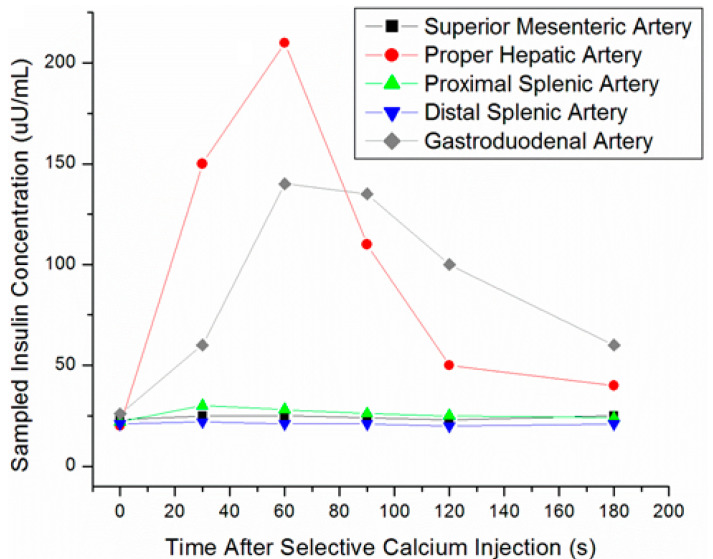
Selective arterial calcium stimulation test (SACST) data from pancreatic head insulinoma. Hepatic venous insulin was markedly elevated after stimulation of either the proper hepatic or gastroduodenal arteries.

SACST is a minimally-invasive procedure usually performed by interventional radiologists, in which arterial stimulation of various portions of the pancreas is paired with venous sampling in order to localize the portion of the pancreas housing the insulinoma. Branches supplying each portion of the pancreas are selected and injected with calcium sequentially. Blood from the hepatic vein is then sampled at time intervals from 10 to 180 s post-calcium injection in each artery. Calcium stimulates release of insulin from hyperfunctioning, abnormal beta-cells found in insulinomas, but not from normally functioning beta-cells of the pancreatic parenchyma [58]. When an artery supplying the insulinoma is stimulated, the corresponding venous blood samples will demonstrate focally increased insulin secretion compared with other regions of the pancreas (Figure 4).

**Figure 5:**
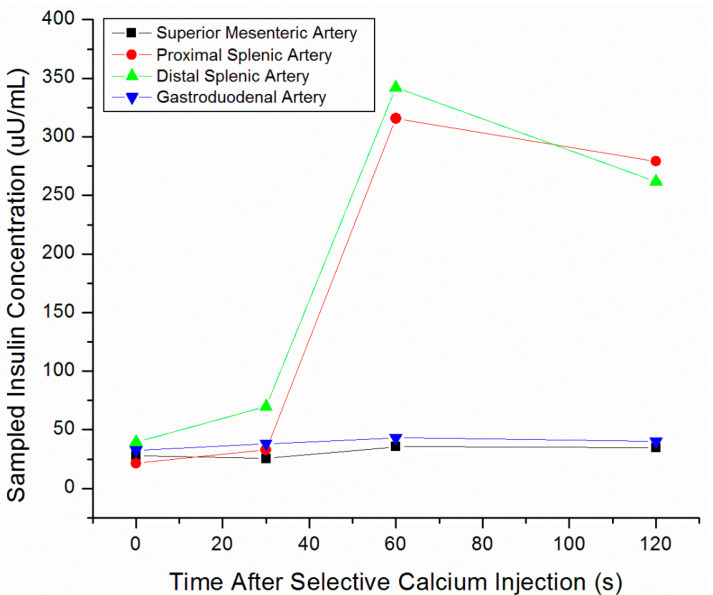
SACST data from pancreatic tail insulinoma. Hepatic venous insulin was markedly elevated after stimulation of either the proximal or distal splenic arteries.

The accuracy of SACST in localizing insulinomas ranges from 67–100%, with most articles reporting ≥90% accuracy [14,16,17,32,33]. SACST is especially useful for localizing tumors in the pancreatic body and tail, which are not as easily seen with EUS (Figure 5).

**Figure 6:**
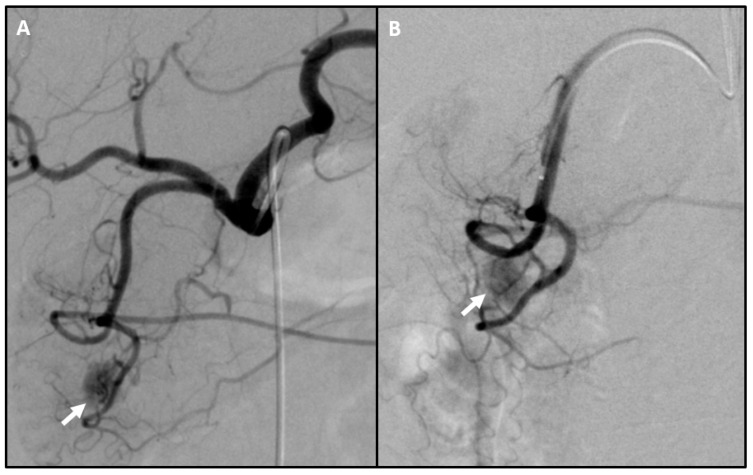
Hypervascular insulinoma on arteriogram. (**A**) Celiac arteriogram after selection with a Sim 1 catheter revealing conventional anatomy and a hypervascular insulinoma (arrow) in the territory of the gastroduodenal artery. (**B**) Sub-selective gastroduodenal arteriogram with a Progreat catheter confirms hypervascular insulinoma (arrow). No corresponding enhancing lesion was present on pre-procedural CT or MRI.

Occasionally, a hypervascular pancreatic tumor corresponding to the insulinoma is visualized angiographically during the procedure (Figure 6). In such cases, the tumor might be amenable to catheter-based embolic therapy or pre-operative methylene blue injection for improved surgical localization [12,59].

Additionally, the SACST can differentiate focal hypersecretion secondary to insulinoma(s) from diffuse hypersecretion in the setting of a non-localized endogenous hyperinsulinemia. [60,61]. Positivity in two non-overlapping arterial distributions increases the likelihood of diffuse hypersecretion. However, this may not reliably differentiate between diffuse hypersecretion and multiple functional insulinomas, the latter of which can be seen in MEN-1 syndrome [62].

## 6. Pancreatic Vascular Anatomy

**Figure 7:**
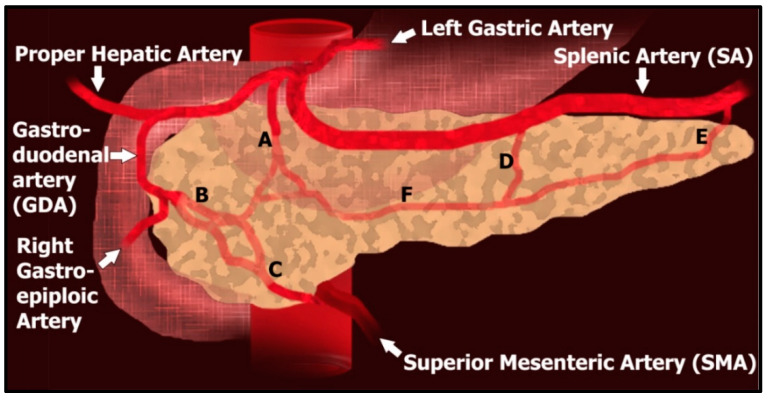
Normal pancreatic arterial supply. The dorsal pancreatic artery (**A**) has a highly variable origin, typically within 2 cm of the celiac trunk, and is often the largest pancreatic artery. The superior pancreaticoduodenal artery (**B**) arises from the gastroduodenal artery (GDA) and supplies the pancreatic head. The inferior pancreaticoduodenal artery (**C**) arises from the superior mesenteric artery (SMA) and supplies the uncinate process. Arising from the splenic artery (SA) is the pancreatica magna artery (**D**), which supplies the body, and more distally, the caudal pancreatic artery (**E**), which supplies the tail. The transverse pancreatic artery (**F**) travels along the body and tail, collateralizing with the other pancreatic vessels.

Review of prior localization imaging studies, including CT and MR, can help delineate relevant anatomy and guide which regions to assess for insulinoma. The pancreas receives regional blood supply from several arteries (Figure 7). The pancreatic head and uncinate process are supplied by an arcade formed by the superior pancreaticoduodenal artery, which arises from the gastroduodenal artery, and the inferior pancreaticoduodenal artery, typically the first branch of the superior mesenteric artery [63,64]. The pancreatic body and tail primarily receive their blood supply from the dorsal pancreatic artery and the pancreatic branches of the splenic artery. The dorsal pancreatic artery (DPA) is the largest of the pancreatic arteries and has a highly variable origin, arising from either the celiac, common hepatic, or splenic artery, typically within 2 cm of the celiac trunk [65,66]. The DPA bifurcates inferiorly into a right and left branch. The right branch anastomoses with the superior pancreaticoduodenal artery. The left branch courses along the inferior border of the pancreas as the transverse pancreatic artery and usually anastomoses in the middle of the gland with the pancreatica magna artery, the largest pancreatic branch of the splenic artery [67]. Pancreatic venous drainage mainly follows the arterial system and drains into the portal vein via either the splenic vein, from the body and tail of the pancreas, or via the superior mesenteric vein, from the pancreatic head’s venous arcade system.

Accurate angiographic anatomic characterization is key, as pancreatic arterial perfusion is variable and can affect interpretation of SACST data [60]. Two clinically relevant vascular configurations are replacement of the DPA to the SMA and celiac stenosis. In replacement of the DPA to the SMA, the SMA may perfuse the entire pancreas. Thus, positive results during SMA selection can lead to the false interpretation that hyperfunctioning cells are solely located in the uncinate process and head; the uncinate process and head are primarily supplied by the SMA in conventional anatomy. In the presence of celiac stenosis, the SMA may supply the entire pancreas via retrograde flow through the GDA and common hepatic artery. As a result, calcium injection into the GDA would not provide localizing information. Rotational cone-beam CT can be used to clarify regional perfusion when uncertain. For example, if retrograde flow through the GDA is suspected, cone-beam CT during the SMA arteriogram can better define the 3D perfusion of the pancreas by the SMA [68,69].

Anomalous venous drainage, while uncommon, may also affect interpretation of SACST data. A clinically relevant example is the presence of splenic varices and a porto-systemic shunt from the splenic vein into a systemic vein, commonly the left renal [60]. This can result in false negative localization during splenic arterial stimulation if venous blood samples are obtained from the hepatic veins, as is standard. Instead, venous blood samples should be obtained from the systemic vein(s) providing primary drainage of the venous shunt.

## 7. Pre-Procedural Workup

The patient should have a biochemical diagnosis of endogenous hyperinsulinemic hypoglycemia secondary to insulinoma. Exogenous insulin administration must be ruled out. Prior imaging should be reviewed to verify that adequate non-invasive imaging has been undertaken in the attempt to localize the tumor. Additionally, prior images may reveal vascular anomalies that may affect SACST localization. Medications which inhibit insulin secretion, such as diazoxide and octreotide, may cause false negatives and must be discontinued prior to SACST.

As per Society of Interventional Radiology guidelines, SACST is considered a low bleeding risk procedure [70]. Routine pre-procedure testing for coagulopathy is not recommended except in cases where patients are suspected to have a bleeding diathesis or thrombocytopenia. Recommended (international normalized ratio) INR thresholds are <1.8 for femoral arterial access and <2.2 for radial arterial access. Platelet count should be >20 × 10^9^/L. Prophylactic antibiotic is typically not necessary as the procedure is considered a clean procedure.

SACST is usually performed with conscious sedation. Equipment for monitoring intraprocedural blood glucose, such as finger stick meters, and dextrose solution for intravenous admission should be available.

## 8. Procedural Description

The SACST is a procedure of moderate technical difficulty and requires an interventional radiologist familiar with microcatheter technique, anatomy, and procedural technique. A fluoroscopic unit capable of digital subtraction angiography of sufficient quality to carefully evaluate visceral anatomy is essential, preferably one capable of rotational cone-beam CT. In addition to standard guidewires and micropuncture sets, the equipment (Table 2) and typical approach for the procedure at our institution are described below. Total disposable equipment cost varies and reimbursement differs based on insurance.

For hepatic venous sampling, the right internal jugular vein is accessed with a micropuncture set using standard Seldinger technique. A 6 Fr vascular sheath secures the access, and a 6 Fr Cobra catheter, one with added sideholes, is advanced into the right hepatic vein. Venous blood samples will be obtained from this catheter.

For selective arterial calcium stimulation, we access either common femoral artery with a micropuncture set using standard Seldinger technique. A 5 Fr vascular sheath secures the access, and a catheter is used to select major visceral arterial branches from the aorta. For initial anatomic characterization, the celiac artery is catheterized and diagnostic angiography performed. At this point, the operator can assess the pancreatic vascular anatomy. On angiography, insulinomas are well-defined round or oval tumors which avidly enhance during the early arterial phase and during a variable length of the venous phase [71]. If variant anatomy is suspected, the SMA should also be catheterized for an angiogram.

Using a microcatheter, the arteries feeding each region of the pancreas are selected sequentially. For typical vascular anatomy, we select the distal splenic artery, proximal splenic artery, proper hepatic artery, gastroduodenal artery, and superior mesenteric artery. Once a vessel is selected, we obtain 5 cc samples of arterial and venous blood prior to injection. We then inject calcium gluconate into the selected artery (5 cc calcium gluconate diluted to 1 mg/kg) and obtain 5 cc samples of blood from the draining hepatic vein 30, 60, 90, 120, and 180 s post-injection. We wait a minimum of 5 min following the injection of calcium gluconate prior to sampling the subsequent vascular territory. A set of 7 samples (1 arterial and 6 venous) should be received for each vessel that is injected with calcium gluconate. For typical pancreatic vasculature and 6 selected arteries, this would result in 42 samples and 210 cc of blood being removed; this blood loss should be factored into final procedural estimated blood loss. The level of insulin is evaluated in each sample of blood. A greater than two-fold increase in the concentration of insulin from pre-injection baseline indicates that an insulinoma is present in the region of the pancreas being injected.

While we are not aware of any reports in the literature, there is a theoretical risk that the patient may develop intraprocedural hypoglycemia. Therefore, peripheral venous blood glucose should be measured periodically during the procedure, and the patient should be lightly sedated so that he or she can report symptoms of hypoglycemia. Dextrose should be readily available in case this complication arises [60].

If desired, assessment for functional hepatic metastatic disease may be performed by cannulation and subsequent calcium stimulation of the right and left hepatic arteries [72]. The presence of stimulated metastatic disease should be accompanied by a rise in sampled venous insulin. Note that venous sampling should be obtained from the ipsilateral hepatic vein after stimulation (e.g., sample from left hepatic vein after stimulating the left hepatic artery).

It is critical to document the position of the catheter tip where the sample was obtained, and the arterial injection performed. This serves as a quality assurance check to confirm accurate calcium delivery and venous sampling. In our experience, the patient will be exposed to 150–250 mGy and approximately 100 cc of contrast during a typical SACST procedure.

## 9. Conclusions

Preoperative localization of insulinomas improves outcomes from surgery. For patients for whom non-invasive testing fails to identify the tumor, SACST may be helpful [6,7]. With an established procedure protocol, knowledge of variant pancreatic arterial anatomy, excellent imaging equipment, and a multidisciplinary approach, SACST can be an effective test and a valuable contribution to patient care.

## Figures and Tables

**Table 1:** Summary of localization techniques.

Method	Detection Rate	Pros	Cons
Transabdominal US [19,20,21]	9–63%	Inexpensive. Widely available.	Limited by body habitus and bowel gas. Poor visualization of pancreatic tail and small tumors.
Contrast Enhanced CT [22,23,24]	63–94%	Rapid imaging. High spatial resolution. Visualizes anatomy and potential metastatic disease.	Ionizing radiation. Iodinated contrast.
Contrast-Enhanced MRI [25,26]	60–90%	No ionizing radiation. No iodinated contrast.	Expensive. Confined space. Prone to motion artifact.
Somatostatin Receptor Imaging [27,28,29,30]	47–60% or 32–90%, per radiotracer	Useful for troubleshooting when other imaging is negative or equivocal.	Ionizing radiation. Long image acquisition time. Poor spatial resolution.
Endoscopic Ultrasound [31]	40–93%, per pancreatic region	Excellent visualization of pancreatic head. May obtain concurrent biopsies.	Invasive. Poor visualization of pancreatic tail.
SACST [14,16,17,32,33]	67–100%	High sensitivity.	Invasive. Ionizing radiation. Requires technical expertise.
Operative Inspection and Palpation [16,17]	77–91%	Highly effective when performed by expert surgeon.	Requires open surgery and operative experience.

**Table 2:** Suggested equipment.

**Hepatic Venous Sampling**
6 Fr short sheath (right internal jugular vein), no flush
6 Fr 65 cm Cobra catheter, with added side hole
42 labeled 10 cc syringes
**Selective Arterial Calcium Stimulation**
5 Fr short sheath (right common femoral artery), normal saline flush
5 Fr 100 cm Sim 1 catheter
130 cm Progreat microcatheter and Fathom microwire
10% calcium gluconate solution (940 mg/10 cc) diluted to 5 cc aliquots of 1 mg/kg (0.025 mEq Ca/kg)
